# Alteration of brain temperature and systemic inflammation in Parkinson’s disease

**DOI:** 10.1007/s10072-019-04217-3

**Published:** 2020-01-10

**Authors:** Hsiu-Ling Chen, Kei Yamada, Koji Sakai, Cheng-Hsien Lu, Meng-Hsiang Chen, Wei-Che Lin

**Affiliations:** 1 Department of Diagnostic Radiology, Kaohsiung Chang Gung Memorial Hospital and Chang Gung University College of Medicine, No. 123, Dapi Rd. Niaosong Dist, Kaohsiung City, 83301 Taiwan Republic of China; 2 Department of Radiology, Kyoto Prefectural University of Medicine, Kajii-cho, Kawaramachi-Hirokoji, Kamigyo-ku, Kyoto, 602-8566 Japan; 3 Department of Neurology, Kaohsiung Chang Gung Memorial Hospital and Chang Gung University College of Medicine, No. 123, Dapi Rd. Niaosong Dist, Kaohsiung City, 83301 Taiwan Republic of China

**Keywords:** Oxidative stress, Diffusion-weighted MRI, Autonomic diseases, Mitochondrial disorders

## Abstract

**Objectives:**

Parkinson’s disease (PD) is known to be related to various factors, including neuroinflammation, increased oxidative stress, and brain temperature alteration. We aimed to evaluate the correlation between these factors using diffusion-weighted imaging (DWI) thermometry and blood tests of systemic inflammation.

**Methods:**

From July 2012 to Jun 2017, 103 patients with PD (44 men and 59 women; mean age, 60.43 ± 9.12 years) and 106 sex- and age-matched healthy volunteers (48 men and 58 women; mean age, 58.16 ± 8.45 years) retrospectively underwent magnetic resonance DWI thermometry to estimate brain intraventricular temperature (*T*_v_). Subjects were divided into three subgroups in light of their ages. The tested inflammatory markers included plasma nuclear DNA, mitochondrial DNA, apoptotic leukocytes, and serum adhesion molecules. The correlations among the *T*_v_ values, clinical severity, and systemic inflammatory markers were then calculated.

**Results:**

The PD patients did not show a natural trend of decline in *T*_v_ with age. Comparisons among the different age groups revealed that the younger PD subjects had significantly lower *T*_v_ values than the younger controls, but the older subjects had no significant group differences. Overall, the PD patients exhibited lower *T*_v_ values than the controls, as well as increased oxidative stress. The brain temperature showed positive correlations with inflammatory markers, including plasma nuclear DNA and L-selectin levels, in all the subjects.

**Conclusions:**

Possible pathophysiological correlations between systemic inflammation and brain temperature were indicated by the results of this study, a finding which may aid us in investigating the underlying pathogenesis of PD.

**Electronic supplementary material:**

The online version of this article (10.1007/s10072-019-04217-3) contains supplementary material, which is available to authorized users.

## Introduction

The pathophysiological basis of the Parkinson’s disease (PD) is the degeneration of dopaminergic neurons in the substantia nigra and the presence of Lewy bodies, which are made of α-synuclein, in those neurons [1]. In PD patients, the activity of mitochondrial complex I, which is one of the components of the electron transport chain, is impaired in various regions, including the substantia nigra, skeletal muscles, and platelets [2]. The structural changes in complex I also make the dopaminergic neurons more susceptible to neurotoxins [3] and results in the chronic production of reactive oxygen species (ROS), increased oxidative stress [4], and subsequent neuroinflammation.

In addition to the motor symptoms in PD, α-synuclein aggregates and Lewy bodies can be seen in autonomic regulatory regions, including the hypothalamus, sympathetic system, and parasympathetic system, and the damage to this circuit is believed to play a central role in the autonomic dysfunctions of these patients [5]. In addition, the brains of patients with mitochondrial diseases have previously been reported to display a protected hypothermic conditions [6] aimed at reducing the oxidative stress and promoting cellular survival [7]. Disturbed mitochondrial function and a disturbed autonomic system [8] will alter the thermal regulation of PD patients [8], especially as the patients grow older or when the disease status progresses.

Recently, neuroinflammation is considered to be one of the major factors in the progression of PD [9], but the role of inflammatory markers, such as apoptotic leukocytes, plasma DNA, and adhesion molecules in peripheral blood, in correlation with the brain temperature has not been well investigated. We hypothesized that the increased circulation of inflammatory markers in patients with PD is correlated with the alteration of brain temperature. Thus, this study aimed to evaluate the brain temperature using diffusion-weighted imaging (DWI) thermometry in order to further explore the pathophysiology of PD. To accomplish this task, the levels of systemic inflammatory markers and brain temperature were investigated in different age groups of patients with PD as well as healthy controls. Furthermore, the relationships between brain temperature and systemic inflammation were investigated.

## Materials and methods

### Participants

This cross-sectional retrospective study targeted patients with idiopathic PD. One hundred and three patients with idiopathic PD (44 men and 59 women; mean age, 60.43 ± 9.12 years), and one hundred and six sex- and age-matched healthy volunteers (48 men and 58 women; mean age, 58.16 ± 8.45 years) were recruited. These participants were enrolled in our previous studies [9–11], and the data were collected from past records. The exclusion criteria for this study included age < 16 or > 80 years, evidence of alcoholism, a history of other neurologic or psychiatric illness potentially affecting the central nervous system, and severe recent life events which could affect the results of the neuropsychiatric or neuroimaging surveys. The protocols of this study were approved by the relevant local ethics committee. Written informed consent was collected from each participant prior to his or her participation in this study.

Idiopathic PD was diagnosed according to the Parkinson’s Disease Society’s criteria by an experienced neurology specialist and only in the event that the given patient had no prior history of other neurologic or psychiatric illnesses and psychotropic medications. Patients with idiopathic PD were treated at the neurology department of a hospital. In each case, the duration of the disease and treatment, and the current daily dosage of levodopa were recorded. The clinical evaluations for disease severity were performed in the “OFF” status. The Unified Parkinson’s Disease Rating Scale (UPDRS) was applied via clinical observation and interview to evaluate multiple aspects of PD. The modified Hoehn and Yahr (H&Y) staging scale was also used to provide an evaluation of functional disability and track the progression of PD. Furthermore, the Schwab and England (S&E) scale was used to record the subjects’ abilities.

All of the subjects were also divided into three subgroups according to their age: subgroup 1, including participants 30–54 years old; subgroup 2, including participants 55–64 years old; and subgroup 3, including participants 65–79 years old.

### Blood sampling and laboratory investigations

Systemic inflammation and oxidative stress were evaluated in some of the subjects in terms of the percentage of apoptotic peripheral leukocytes, the plasma levels of nuclear DNA, mitochondrial DNA, and serum adhesion molecules. Blood samples were collected from some of the patients with PD and some of the controls by venipuncture of forearm veins on the same day as the MRI study and neuropsychological testing. Specifically, 99 patients with PD and 59 controls underwent blood sampling. Thus, the further statistical analyses and graph plots related to inflammatory markers were done with this limited number of PD patients and controls. The procedural details were as described previously [9, 10, 12], and are summarized in the supplementary material.

Leukocytes and their subtypes were identified based on the intensity of CD45 expression. Apoptotic cells were defined as those that were positive for APO 2.7.

The plasma nuclear DNA was measured by a real-time quantitative polymerase chain reaction (RT-PCR) assay (Roche LightCycler, Roche, Grenzach-Wyhlen, Germany) for the β-globin and ND2 genes as plasma nuclear and mitochondrial DNA.

Serum adhesion molecules, including serum ICAM-1, VCAM-1, E-selectin, L-selectin, and P-selectin levels were assessed using commercially available enzyme-linked immunosorbent assays (R&D Systems, Minneapolis, MN, USA).

### MR imaging

#### Data acquisition

The MRI scan of each participant was performed using a 3.0-Tesla whole-body GE Signa MRI system (General Electric Healthcare, Milwaukee, WI, USA) equipped with an eight-channel head coil. DTI was conducted along the anterior-posterior commissure line (AC-PC line) in the axial plane using a single-shot spin-echo echoplanar imaging (EPI) sequence. The DTI gradient encoding schemes included 13 noncollinear directions with a *b* value of 1000 s/mm^2^ and a nondiffusion-weighted image volume (*b* value 0 s/mm^2^).

#### Temperature estimation

The kinetic theory states that a direct relationship exists between the absolute temperature and the diffusion coefficient. The diffusion coefficient of non-restricted water molecules can be reliably measured using MRI. Studies by Mills and Kozak et al. have shown that the cerebrospinal fluid temperature can be estimated using this relationship [13, 14]^.^ Based on previous studies, we calculated the diffusion constant using the following equation:

$$ D=\ln\ \left({S}_0/S\right)/b $$

where *D* is the diffusion constant (mm^2^/s), *b* is the applied diffusion weighting (s/mm^2^), and *S*_0_ and *S* are the voxel signal intensities of the reference and DWIs, respectively. The *D* value was converted to the corresponding temperature by the following equation:

$$ T=2256:74/\ln\ \left(4.39221/D\right)-273.15 $$

where *T* is the temperature (°C). The temperature estimation was only considered within the lateral ventricles, since this method is only applicable to nonrestricted water. This DWI-based MR thermometry of the ventricles was calculated using an automated method developed by Sakai et al. [15].

### Statistical analyses

#### Analysis of demographic data between groups

The statistical analysis was performed using the Statistical Package for Social Sciences (SPSS) software package (version 17, SPSS Inc. Chicago, IL, USA). Age and sex were compared between the study groups by the independent *t* test and Pearson chi-square test. An analysis of covariance (ANCOVA) model with age and gender as covariates was used to determine group differences in the oxidative stress parameters in terms of the percentage of apoptotic peripheral leukocytes and the plasma levels of nuclear DNA, mitochondrial DNA, and serum adhesion molecules. Statistical significance was set at *p* < 0.05.

#### Analysis of brain temperature between groups

ANCOVA with age and gender as potential confounding variables was used to compare the PD and control groups in terms of intraventricular brain temperature (*T*_v_). ANCOVA with age as the covariant was used to compare the group differences of *T*_v_ between the PD and control groups in the male and female populations, respectively. ANCOVA with gender as the covariant was used to compare the group differences of *T*_v_ among the three age subgroups. Statistical significance was set at *p* < 0.05.

#### Correlations among brain temperature, clinical severity, and inflammatory parameters

Partial correlation analysis adjusted for age and gender was performed with respect to the clinical severity, inflammatory parameters, and *T*_v_. The threshold for statistical significance was set at *p* < 0.05 with a Bonferroni correction accounting for multiple regions of interest comparisons.

## Results

### Demographic characteristics of the participants and the disease severity of the PD patients

The demographic characteristics of the 103 PD cases and 106 controls are listed in Table 1. There were no significant differences in age and sex between the two groups (age: *p* = 0.064; gender: *p* = 0.781).

**Table 1:** Demographic characteristics of patients with PD and control subjects

Variable	PD	Normal	*p* value
Number	103	106	
Gender (M/F)	44/59	48/58	0.781
Age (year)	60.43 ± 9.12	58.16 ± 8.45	0.064
Male	60.16 ± 11.01	57.81 ± 9.88	0.284
Female	60.63 ± 7.51	58.45 ± 7.12	0.110
Disease duration (year)	3.10 ± 3.31		
Treatment duration (month)	16.12 ± 20.10		
Current daily dose (mg)	424.04 ± 402.90		
UPDRS I	3.17 ± 2.55		
UPDRS II	10.24 ± 7.64		
UPDRS III	24.70 ± 16.19		
UPDRS 176	37.23 ± 24.28		
Modified H&Y stage	2.10 ± 1.11		
S&E scale	82.60 ± 18.24		
Inflammatory parameters	*n* = 99	*n* = 59	
Apoptotic neutrophils (%)	1.11 ± 0.89	0.74 ± 0.56	0.005*
Apoptotic monocytes (%)	5.30 ± 6.30	2.97 ± 4.05	0.012*
Apoptotic lymphocytes (%)	0.73 ± 0.52	0.49 ± 0.40	0.002*
Total apoptotic leukocytes (%)	1.73 ± 1.20	1.13 ± 0.74	0.001*
Nuclear DNA (ng/mL)	32.69 ± 26.36	19.99 ± 14.41	0.002*
Mitochondrial DNA (ng/mL)	42.51 ± 36.34	51.41 ± 120.47	0.393
ICAM-1	207.77 ± 77.39	213.09 ± 59.46	0.734
VCAM-1	738.10 ± 286.68	640.92 ± 143.86	0.025*
P-selectin	96.30 ± 21.17	84.47 ± 20.58	0.001*
L-selectin	860.98 ± 175.11	955.84 ± 218.81	0.008*
E-selectin	36.53 ± 18.53	34.54 ± 14.81	0.526

Data are presented as mean ± standard deviation. Age data were compared by independent *t* test. Gender data were compared by Pearson chi-square test. The inflammatory parameters data were compared by analysis of covariance (ANCOVA) after controlling for age and gender

*UPDRS*, Unified Parkinson Disease Rating Scale; *H&Y stage*, Hoehn and Yahr stage; *S&E scale*, Schwab and England scale

*p < 0.05

### Comparison of inflammatory parameters between groups

The percentages of total apoptotic leukocytes and their subsets, including neutrophils, monocytes, and lymphocytes, were significantly higher in the PD group than in the controls. The PD patients exhibited higher nuclear DNA levels (*p* = 0.002), VCAM-1 levels (*p* = 0.002), and P-selectin levels (*p* = 0.001), as well as lower L-selectin levels (*p* = 0.008), compared with the controls.

### Comparison of brain temperature in groups and subgroups

**Fig. 1:**
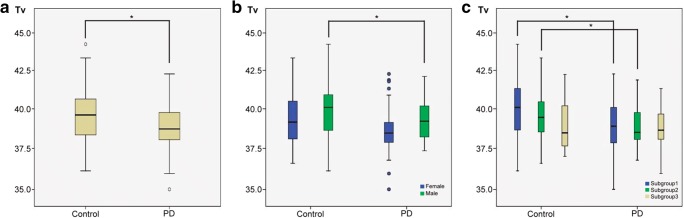
**a** Measured brain intraventricular temperatures (*T*_v_) in patients with PD (*n* = 103) compared with healthy controls (*n* = 106). The *T*_v_ values of the patients with PD were significantly lower than those of the controls (*p* = 0.006) after controlling for age and gender. **b** Measured *T*_v_ in male and female patients with PD (male, *n* = 44; female, *n* = 59) compared with male and female healthy controls (male, *n* = 48; female, *n* = 58), respectively. The *T*_v_ values of the male patients with PD were significantly lower than those of the male controls (*p* = 0.039) after controlling for age. **c** Measured *T*_v_ in different age subgroups of patients with PD (subgroup 1, 30–54 years old, *n* = 24, *T*_v_ = 39.01 ± 1.72 °C; subgroup 2, 55–64 years old, *n* = 37, *T*_v_ = 38.83 ± 1.20 °C; subgroup 3, 65–79 years old, *n* = 41, *T*_v_ = 38.88 ± 1.19 °C; *p* = 0.273) compared with different age subgroups of healthy controls (subgroup 1, *n* = 30, *T*_v_ = 40.12 ± 1.90 °C; subgroup 2, *n* = 54, *T*_v_ = 39.52 ± 1.52 °C; subgroup 3, *n* = 22, *T*_v_ = 39.03 ± 1.52 °C; *p* = 0.072), respectively. In the age subgroups of the controls, the *T*_v_ values of subgroup 1 were significantly higher than those of subgroup 2 (*p* = 0.031) and subgroup 3 (*p* = 0.027). The *T*_v_ values of subgroups 1 and 2 patients with PD were significantly lower than those of the controls (*p* = 0.042 and 0.024) after controlling for gender

The box and whisker plot of measured *T*_v_ in the two groups is shown in Fig. 1. The *T*_v_ of the patients with PD was lower than that of the controls (*p* = 0.006). In the male population, the *T*_v_ in the patient group was slightly lower than that in the control group (*p* = 0.039). In the female population, the difference in *T*_v_ between the patient and control groups did not reach a statistically significant level (*p* = 0.052).

Among the different age subgroups of the PD patients, there was no significant difference in *T*_v_ (*p* = 0.273). Among the different age subgroups of the controls, the *T*_v_ of subgroup 1 was significantly higher than those of subgroup 2 (*p* = 0.031) and subgroup 3 (*p* = 0.027). In the comparison between the PD and controls, the *T*_v_ values of the subgroup 1 and 2 PD patients were significantly lower than those of the controls (*p* = 0.042 and *p* = 0.024).

### Relationships between brain temperature and inflammatory parameters

**Fig. 2:**
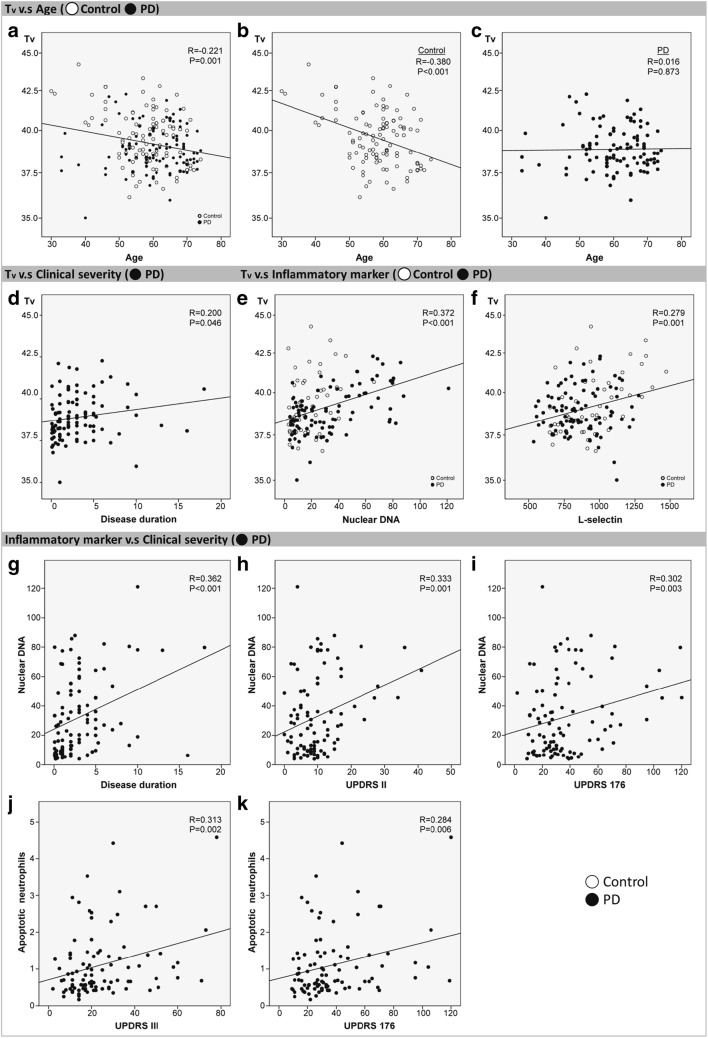
Correlations among age, clinical severity, inflammatory markers, and intraventricular temperature. The line indicates the result of linear fitting. Intraventricular temperature in all subjects (**a**) and controls (**b**) tended to decrease with age, with a significant correlation seen after controlling for gender. There was no correlation between age and *T*_v_ in the PD patients (**c**) after controlling for gender. The *T*_v_ was positively correlated with disease duration (**d**), nuclear DNA (**e**), and L-selectin (**f**) after controlling for age and gender. The level of plasma nuclear DNA revealed statistically positive correlations with disease duration (**g**), UPDRS II (**h**), and UPDRS 176 (**i**). The percentage of apoptotic neutrophils revealed statistically positive correlations with UPDRS III (**j**) and UPDRS 176 (**k**)

The results of the correlation analysis between brain temperature and inflammatory parameters are also presented in Table 2 and Fig. 2e, f. Both the plasma nuclear DNA level and L-selectin level revealed statistically positive correlations with *T*_v_ (*p* < 0.001 and *p* = 0.001) in all the subjects. Meanwhile, the plasma nuclear DNA level revealed a significant positive correlation with *T*_v_ (*p* < 0.001) in the PD group, but no statistical correlation (*p* = 0.071) in the control group. In contrast, the L-selectin level revealed a significant positive correlation with *T*_v_ (*p* = 0.005) in the control group, but no statistical correlation (*p* = 0.275) in the PD group.

**Table 2:** Correlations among brain intraventricular temperature, clinical severity, and systemic inflammatory parameters

Variables	*T*_v_	
	*R*	*p*
Clinical data		
Disease duration	0.200	0.046*
Treatment duration	0.095	0.345
Current daily dose	0.031	0.761
UPDRS I	0.157	0.120
UPDRS II	0.136	0.181
UPDRS III	0.074	0.468
UPDRS 176	0.109	0.284
Modified H&Y stage	0.141	0.165
S&E scale	− 0.197	0.052
Inflammatory parameters		
Apoptotic neutrophils	− 0.138	0.090
Apoptotic monocytes	− 0.062	0.444
Apoptotic lymphocytes	− 0.018	0.826
Total apoptotic leukocytes	− 0.131	0.108
Nuclear DNA	0.372	< 0.001*
VCAM-1	− 0.054	0.520
P-selectin	− 0.021	0.299
L-selectin	0.279	0.001*

Partial correlation analysis adjusted for age and gender was performed to correlate the *T*_v_ with the clinical severity and inflammatory parameters

*p < 0.0, threshold for statistical significance

### Relationship between brain temperature and age

Correlation analysis was used to evaluate the relationship between age and brain temperature in the different groups (Fig. 2a–c. A significant negative correlation was evident between the *T*_v_ and age in all the subjects (*R* = − 0.221; *p* = 0.001) and in the control group (*R* = − 0.380, *p* < 0.001). However, the *T*_v_ revealed no statistically significant correlation with age in the PD group (*R* = 0.016, *p* = 0.873).

### Relationship between brain temperature and clinical severity

Correlation analysis was used to evaluate the relationship between clinical severity and brain temperature in the PD group (Table 2; Fig. 2d). The *T*_v_ revealed a significant positive correlation with disease duration (*p* = 0.046).

### Relationships between clinical severity and inflammatory parameters

Correlation analysis was used to evaluate the relationship between clinical severity and inflammatory markers in the PD group (Table 3; Fig. 2g–k). The percentage of apoptotic neutrophils revealed statistically positive correlations with UPDRS III (*p* = 0.002) and UPDRS 176 (*p* = 0.006). The level of plasma nuclear DNA revealed statistically positive correlations with disease duration (*p* < 0.001), UPDRS II (*p* = 0.001), and UPDRS 176 (*p* = 0.003).

**Table 3:** Correlations between clinical severity and systemic inflammatory parameters

Variables	Apoptotic neutrophils		Apoptotic monocytes		Apoptotic lymphocytes		Total apoptotic leukocytes		Nuclear DNA		VCAN-1		P-selectin		L-selectin	
	*R*	*p*	*R*	*p*	*R*	*p*	*R*	*p*	*R*	*p*	*R*	*p*	*R*	*p*	*R*	*p*
Disease duration	0.056	0.594	0.017	0.870	− 0.059	0.572	0.066	0.533	0.362	< 0.001*	− 0.014	0.895	0.119	0.248	0.078	0.448
Treatment duration	0.057	0.587	0.091	0.387	0.262	0.011	0.206	0.048	− 0.068	0.513	0.245	0.016	− 0.257	0.011	− 0.167	0.103
Current daily dosage	0.097	0.353	− 0.005	0.965	− 0.062	0.557	0.124	0.238	0.154	0.133	0.205	0.046	0.004	0.967	0.042	0.685
UPDRS I	0.157	0.138	− 0.111	0.297	− 0.132	0.214	0.089	0.404	0.220	0.033	0.009	0.930	0.061	0.562	− 0.086	0.411
UPDRS II	0.203	0.053	0.022	0.837	− 0.041	0.699	0.201	0.056	0.333	0.001*	0.115	0.269	0.198	0.056	0.060	0.568
UPDRS III	0.313	0.002*	− 0.027	0.799	− 0.084	0.431	0.227	0.031	0.270	0.009	0.171	0.098	0.083	0.428	0.065	0.532
UPDRS 176	0.284	0.006*	− 0.022	0.832	− 0.081	0.445	0.219	0.037	0.302	0.003*	0.148	0.153	0.121	0.244	0.052	0.619
Modified H&Y stage	0.229	0.029	− 0.067	0.530	− 0.138	0.194	0.156	0.140	0.257	0.012	0.192	0.064	− 0.052	0.617	0.029	0.779
S&E scale	− 0.248	0.018	0.054	0.611	0.090	0.394	− 0.156	0.140	− 0.255	0.013	− 0.105	0.312	0.008	0.943	0.040	0.700

Partial correlation analysis adjusted for age and gender was performed to correlate the inflammatory parameters with the clinical severity

*p < 0.05, threshold for statistical significance with a Bonferroni correction accounting for multiple region of interest comparisons

## Discussion

The regulation of brain temperature is dependent on various factors, including cerebral metabolism, cerebral blood flow, and body core temperature. Heat production in the brain is closely related to the cerebral metabolic rates of glucose and oxygen, and the age-dependent decrease in metabolism can lead to temperature decline (Figs. 1c and 2b) [16]. A natural decline in *T*_v_ among normal subjects have been repeatedly shown, but the PD subjects failed to reveal this descent in *T*_v_. Another characteristic finding was that PD patients tended to have lower temperature compared with controls, which was especially evident in the younger age subgroups (Figs. 1c and 2c). For the older subjects (subgroup 3, 65–79 years old), the temperature difference was not significant (Fig. 1c).

Significantly higher levels of circulating apoptotic leukocytes, nuclear DNA, VCAM-1, and P-selectin in PD patients were shown in the present study. These markers are important indicators supporting the presence of underlying systemic inflammation (Table 1). Those markers were also associated with increased disease severity, suggesting that they may play critical roles in the pathophysiology of PD (Table 3; Fig. 2g–k). It is well known that in brain tissue, dysfunction of the blood-brain barrier accompanied by peripheral immune cell infiltration/invasion leads to the loss of dopaminergic neurons caused by programmed cell death [17].

**Fig. 3:**
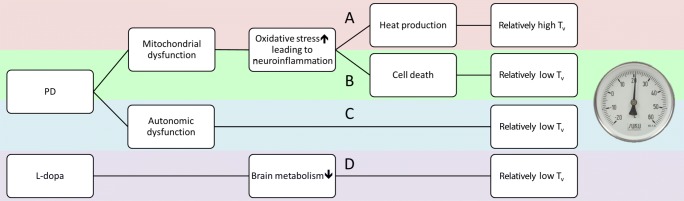
The pathophysiologic model involving oxidative stress and thermal regulation in PD in this study. Mitochondria are responsible for maintaining the cellular energy reserves with heat production. Mitochondrial dysfunction is involved in activated neuroinflammation and increased oxidative stress. The injury from oxidative stress might induce cell apoptosis or death, which would in turn result in the loss of heat production and the associated relatively low *T*_v_ (pathway B). However, in the condition of cell damage but survival, the resulting hyper-metabolism under oxidative stress would promote heat generation and the associated relatively high *T*_v_ (pathway A). The dynamic regulation of pro- and anti-inflammatory cytokines could potentially promote neuroprotection and lead to the initiation of thermal modulation. Other etiologies, such as autonomic dysfunction and dopamine agonists, might also be related to thermal dysregulation in PD (pathways C and D)

This study is the first to report an association between brain temperature and circulating inflammatory markers in PD patients. As the temperature of the brain should be determined by multiple different factors, a simplified summary of those factors is provided in Fig. 3. Heat in the brain is produced mostly by mitochondrial oxidative chemical reactions [6]. As PD patients have impaired mitochondrial biogenesis (mitobiogenesis), this would directly affect the brain temperature of PD patients, and it is thought to be one of the major causes for temperature decline.

Oxidative stress and neuroinflammation also play an important role in determining brain temperature, as they can either elevate or lower it (Fig. 3a, b). Mitochondrial ROS is known to activate signaling pathways that control the initiation of cell death [18]. Increased plasma nuclear DNA is one of the inflammatory markers, and in this study, it was found to have positive correlations with higher brain temperature, disease duration (Fig. 2g), and UPDRS scores (Fig. 2i–k).

Mitochondrial and plasma membranes are the most temperature-sensitive cellular elements. Hyperthermia can further potentiate the cytotoxic effects of ROS into a vicious cycle [19]. In contrast, hypothermia is known to reduce superoxide anion production [20], in addition to preventing hydroxyl radical formation. The injury from oxidative stress might induce cell apoptosis or death, which would result in the loss of heat production and associated hypothermia (Fig. 3, pathway B). However, in the condition of cell damage but survival, the hyper-metabolism under oxidative stress would promote heat generation and associated hyperthermia (Fig. 3, pathway A).

The dynamic regulation of pro- and anti-inflammatory cytokines is suggested to be potentially useful in promoting neuroprotection [21], and it could lead to the initiation of thermal modulation. Since the alteration of temperature and the mechanisms underlying the relationship between oxidative stress and thermoregulation are complex, further studies of animal models might be required to fully clarify the causal relationships among those factors. Other etiologies, such as autonomic dysfunction and dopamine agonists [8], might also play some role in thermal dysregulation (Fig. 3, pathways C and D). Both of them were known to topographically involve similar temperature regulation area and should be further evaluated in the future.

Even though there are many mechanisms to regulate the human core temperature (Fig. 3), PD is ultimately an irreversible neurodegenerative disease. Therefore, the overall *T*_v_ in the PD patients presented in our study was lower than that of the normal controls. However, overlaps in *T*_v_ between the PD patients and the controls were observed. This might have been due to the combined results of cell inflammation with different degrees of the disease in the PD patients, as well as other variations among the physiological effects on the individual subjects, such as variations in the effects on their individual metabolic rates. The higher oxidative stress in the PD patients seen in our results might reflect both anti- and pro-inflammatory processes in their early disease [22] that lead to the overlap with the normal controls in *T*_v_ presentation. As the disease progresses, more oxidative stress induced in neurons undergoing pro-inflammatory processes leads to cell apoptosis, which in turn eventually causes a drop in the overall central temperature. The trajectory of oxidative stress seen in the present study was similar to the trajectory presented in a previous report [23] about ramified microglia transforming into anti-inflammatory and pro-inflammatory phenotypes. However, we could not verify the phenotypes of oxidative stress in our results, so a further validation study should therefore be conducted.

Although this study has yielded useful findings, the interpretation of its results must be done carefully. First, it should be noted that the brain temperature is dependent on the body core temperature, but this latter temperature was not recorded as this was a retrospective study. Second, the ventricular temperatures of all the participants were slightly higher than expected, possibly because the pulsatile movement of the cerebrospinal fluid led to the overestimation of these temperature levels. In any case, the method used was thus only applicable for comparison between the groups. Whether this technique can also be applied to monitor a patient for longitudinal follow-up remains to be tested in future studies. Third, the effects of medication on brain temperature remain unknown. Quite naturally, almost all of the PD patients were undergoing treatment with various medications depending on their clinical symptoms. Last but not the least, the individuals in this group of PD patients were at multiple different stages of disease progression, and therefore, the data used in this study do not constitute an ideal set of data to elucidate the true nature of disease progression. This study was a cross-sectional one, which limits the degree to which its results can be interpreted as representing the full picture of PD. Thus, the causal relationships between inflammation and the alteration of brain temperature in PD still remain to be elucidated. It is hoped, nonetheless, that this study can serve as a basis for further research.

The results of this study support our hypothesis that increased inflammation in patients with PD correlates with the alteration of brain temperature and provide a possible pathophysiological explanation for the associated systemic inflammation and thermoregulation. This model could assist us in investigating the underlying pathogenesis of PD in future research.

## Electronic supplementary material

ESM 1 (DOCX 49 kb)

## Abbreviations

ANCOVA: Analysis of covariance
*T*_v_: Brain intraventricular temperature
DWI: Diffusion-weighted imaging
H&Y: Modified Hoehn and Yahr scale
PD: Parkinson’s disease
ROS: Reactive oxygen species
S&E: Schwab and England scale
UPDRS: Unified Parkinson’s Disease Rating Scale
