# Supplementary material for: Alteration of brain temperature and systemic inflammation in Parkinson’s disease
Source: Neurol Sci. 2020 Jan 10;41(5):1267–76. doi: 10.1007/s10072-019-04217-3 (PMC7196953; doi:10.1007/s10072-019-04217-3)
Supplement: Supplementary file 1 — (DOCX 49 kb) [file 10072_2019_4217_MOESM1_ESM.docx]

**Additional Files**

**Blood Sampling and Laboratory Investigations**

**1. Assessment of Leukocyte Apoptosis**

Procedural details were as described previously [[1](#_ENREF_1),[2](#_ENREF_2)] and are summarized in the supplementary material. A whole blood sample (100 μL) was stained with 10 μL CD45-phycoerythrin (PE)-Cy5 (clone J33; Immunotech, Marseille, France) for 15 min at room temperature while protected from light. The CD45-PE-Cy5 antibody reacted with the CD45 family of transmembrane glycoproteins expressed on the surface of all human leukocytes and was a pan-leukocyte marker. Cells were fixed with 5.5% formaldehyde and washed. Then during this stage, the cells were brought into contact with APO 2.7-PE (clone 2.7A6A3; Immunotech) to determine intracellular antigens. The APO 2.7-PE antibody reacted with a 38 kDa mitochondrial membrane protein (7A6 antigen) that was detectable in nonpermeabilized cells during the late apoptotic state. Mouse immunoglobulin G (IgG)-PE was used as a control for nonspecific staining. The leukocytes were then analyzed by flow cytometry.

Flow cytometry analysis was performed immediately after staining with an Epics XL flow cytometer (Beckman Coulter, USA) using EXPO32 ADC software. Leukocytes and their subtypes were identified by CD45-PE-Cy5-positive and sidescatter gating. A minimum of 5,000 events was collected for total leukocytes in every blood sample. Leukocyte subtypes were identified based on the intensity of CD45 expression. Results were expressed as a percentage of specific fluorescencepositive cells. Apoptotic cells were defined as those that were positive for APO 2.7. A database coordinator monitored all data collection and entry, both of which were checked for any inconsistencies.

**2. Determination of Plasma Nuclear DNA and Mitochondrial DNA Levels.**

In every patient, 3mL of peripheral venous blood was collected into ethylenediaminetetraacetic acid-containing tubes. Procedural details were as conducted previously [[3](#_ENREF_3)] and are summarized in the supplementary material. To ensure cell-free specimen collection, the blood samples were initially centrifuged and the plasma was transferred to clear polypropylene tubes and centrifuged. A QIAamp Blood Kit (Qiagen, Hilden, Germany) was used for DNA extraction and calculating the target DNA concentration according to the manufacturer’s blood and body fluid protocol. The plasma nuclear DNA was measured by a realtime quantitative polymerase chain reaction (RT-PCR) assay (Roche LightCycler, Roche, Grenzach-Wyhlen, Germany) for the β-globin and ND2 genes as plasma nuclear and mitochondrial DNA. Continuous measurements of Sybr green fluorescent dye bound to double-stranded DNA generated in quantitative RT-PCR were done as expression of plasma DNA. The DNA standard curve was generated using human genomic DNA (Roche). Quantitative results are expressed as ng/mL.

**3. Assessment of Serum Adhesion Molecules**

Serum ICAM-1, VCAM-1, E-selectin, L-selectin, and P-selectin levels were assessed using commercially available enzyme-linked immunosorbent assays (R&D Systems, Minneapolis, MN, USA) as previously described [[1](#_ENREF_1)] and are summarized in the supplementary material. In these assays, standards, controls, and unknown samples were incubated in microtitration wells coated with marked (i.e., anti-ICAM-1, VCAM-1, P-selectin, E-selectin, and L-selectin) antibodies. After incubation and washing, the wells were treated with another antiantigen detection antibody labeled with enzyme horseradish peroxidase and they were further incubated with substrate tetramethylbenzidine. An acidic stopping solution was then added and the degree of enzymatic turnover of the substrate was determined by a dual-wave length absorbance measurement at 450 and 620 nm. Absorbance was directly proportional to the concentration of antigens present. A set of standard antigen was used to plot a standard curve of absorbance versus antigen concentration from which the antigen concentrations of the unknowns were calculated.

**References**

1. Chen HL, Lu CH, Lin HC, et al. White matter damage and systemic inflammation in obstructive sleep apnea. Sleep. 2015;38:361-70.

2. Lin WC, Tsai NW, Huang YC, et al. Peripheral leukocyte apoptosis in patients with Parkinsonism: Correlation with clinical characteristics and neuroimaging findings. Biomed Res Int. 2014;2014:635923.

3. Yu CC, Chen MH, Lu CH, et al. Altered Striatocerebellar Metabolism and Systemic Inflammation in Parkinson's Disease. Oxid Med Cell Longev. 2016;2016:1810289.
